# Supplementary material for: Romanian Bee Product Analysis: Chemical Composition, Antimicrobial Activity, and Molecular Docking Insights
Source: Foods. 2024 May 8;13(10):1455. doi: 10.3390/foods13101455 (PMC11119262; doi:10.3390/foods13101455)
Supplement: Supplementary file 1 [file foods-13-01455-s001.zip › foods-2953244-supplementary.pdf]

Supplemmentary files - Antimicrobian activity

Table 2 presents the antimicrobial activity as bacterial inhibition rate (BIR%) values. The supplementary files present the results in graphical form: the bacterial growth rate (BGR%) and bacterial inhibition rate (BIR%) for each strain (Figures S1-S15).

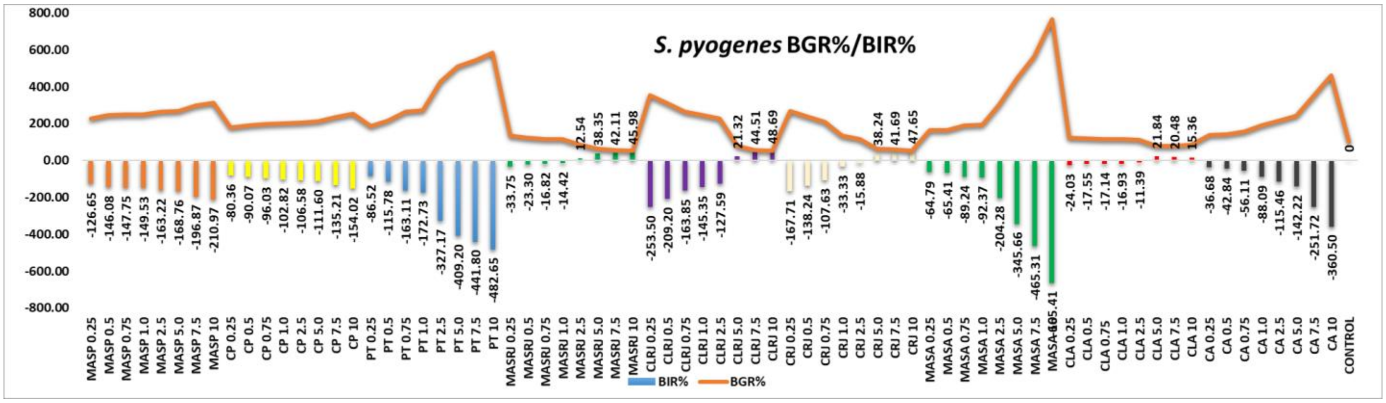

Figure S1. Antibacterial activity against the *S. pyogenes* strain.

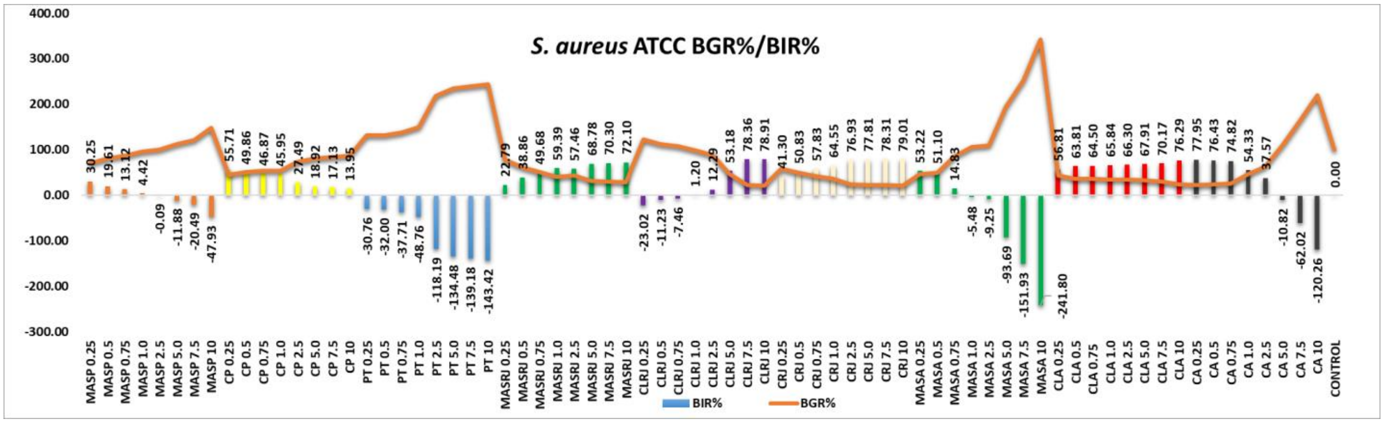

Figure S2. Antibacterial activity against the *S. aureus* strain.

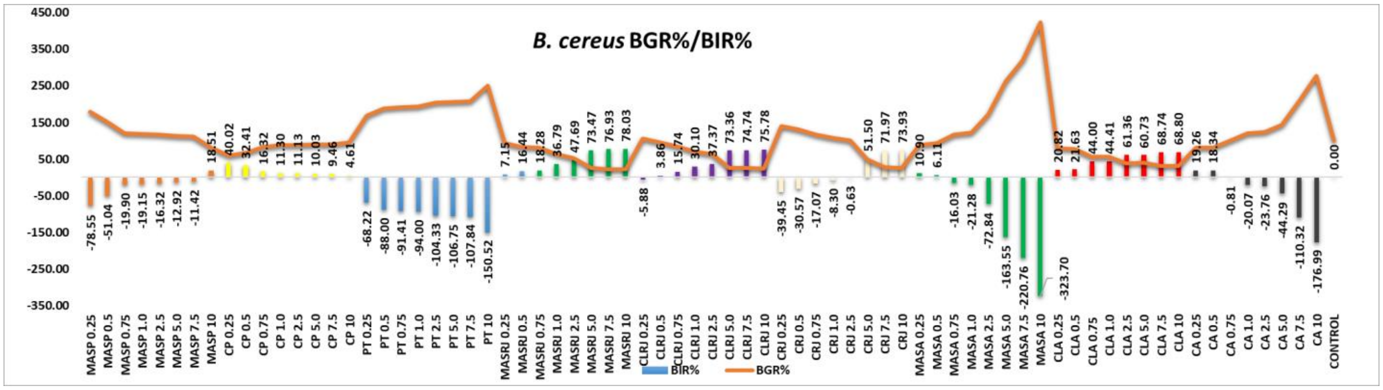

Figure S3. *B. cereus* - Graphical representation of the BGR% and BIR% values.

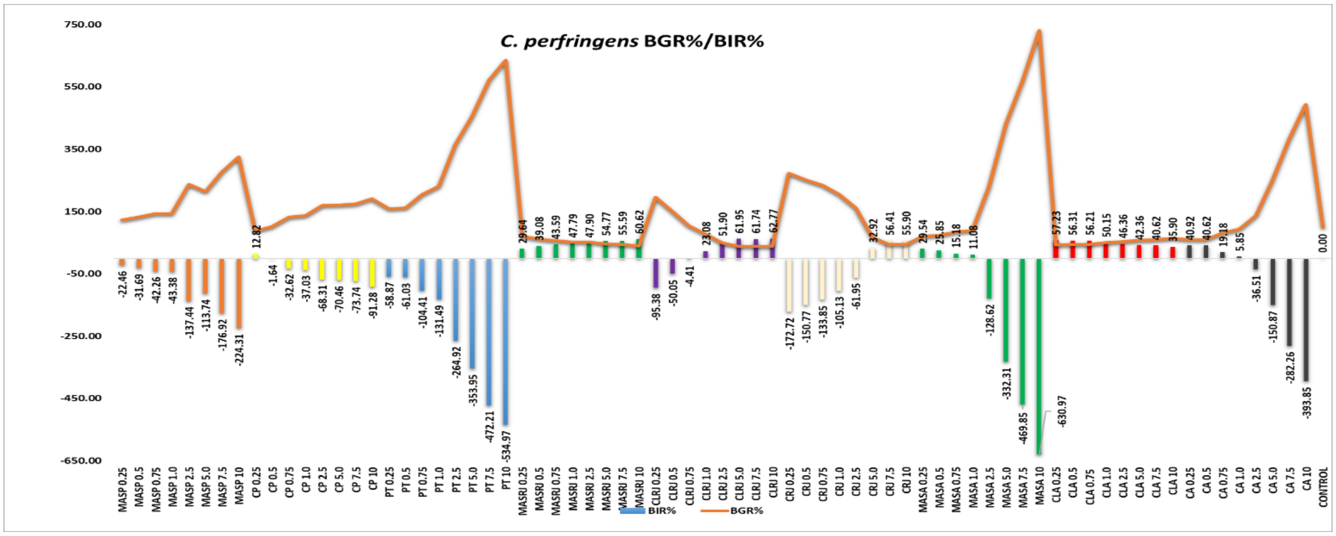

Figure S4. *C. perfringens* - Graphical representation of the BGR% and BIR% values.

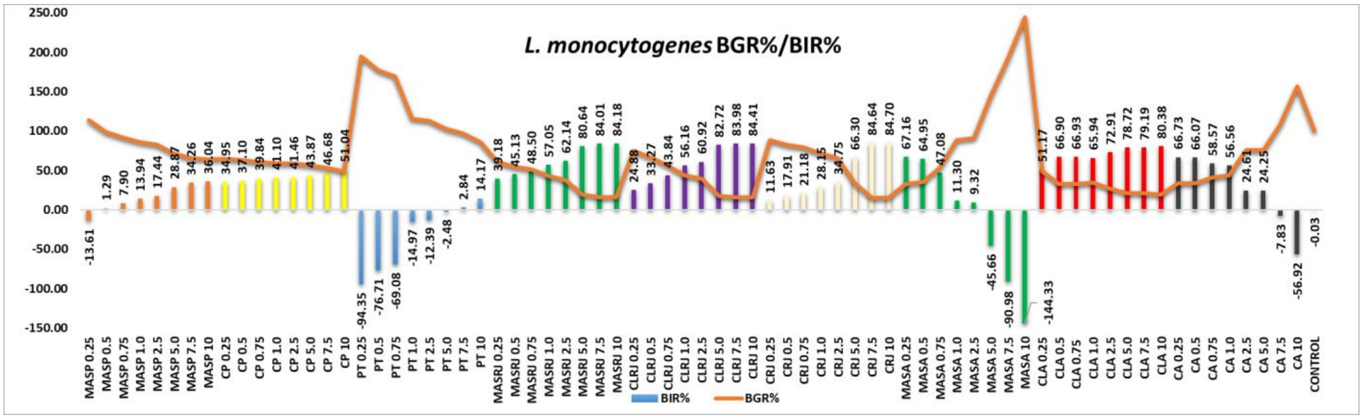

Figure S5. *L. monocytogenes* - Graphical representation of the BGR% and BIR% values.

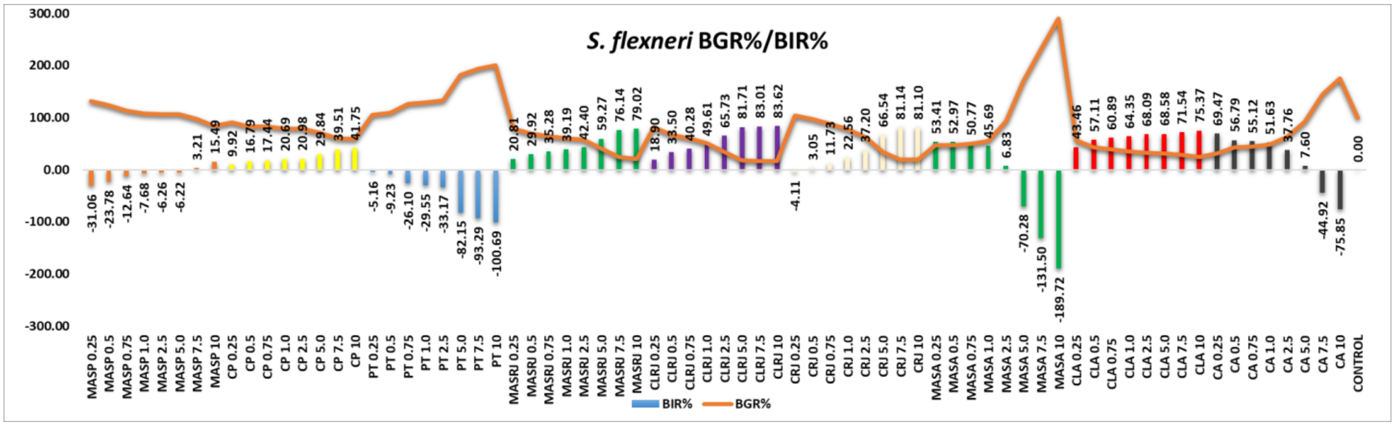

Figure S6. Antibacterial activity against the *S. flexneri* strain.

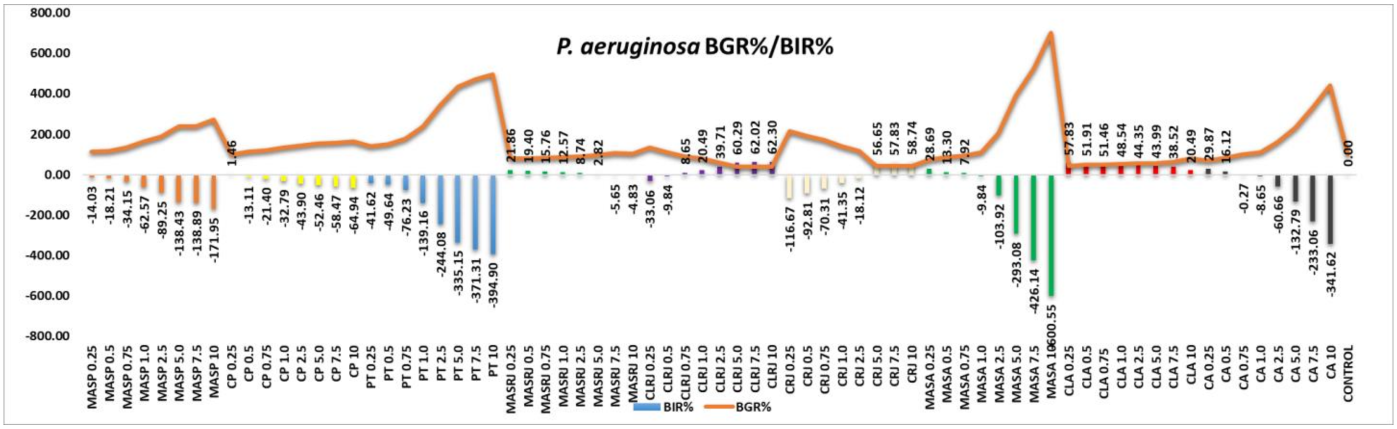

Figure S7. Antibacterial activity against the *P. aeruginoasa* strain.

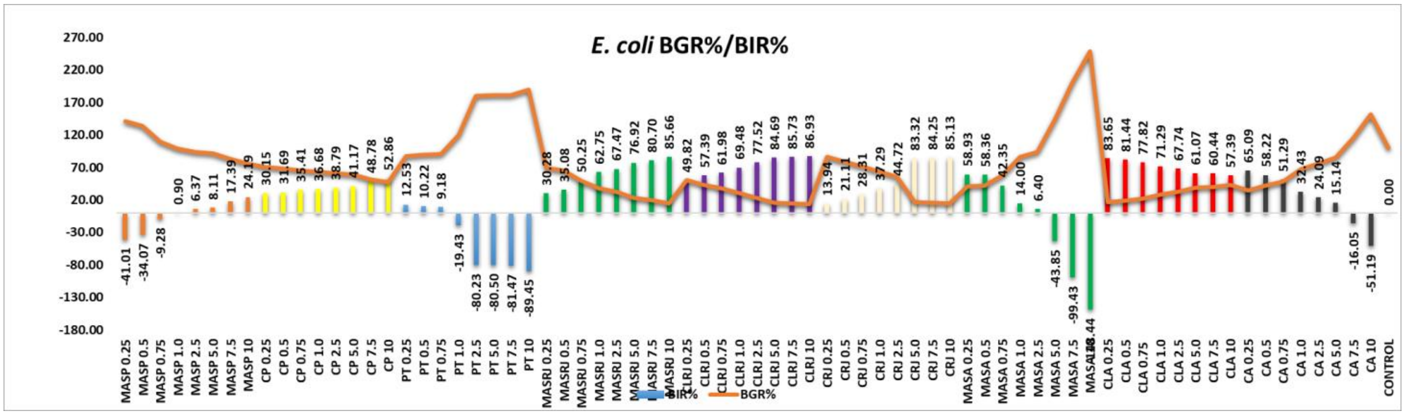

Figure S8. Antibacterial activity against the *E. coli* strain.

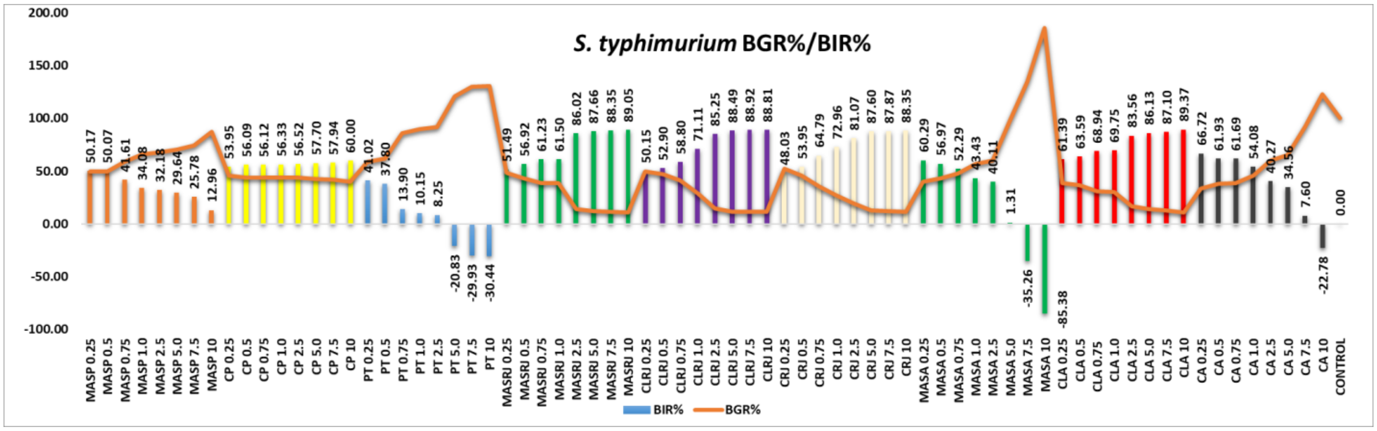

Figure S9. Antibacterial activity against the *S. typhimurium* strain.

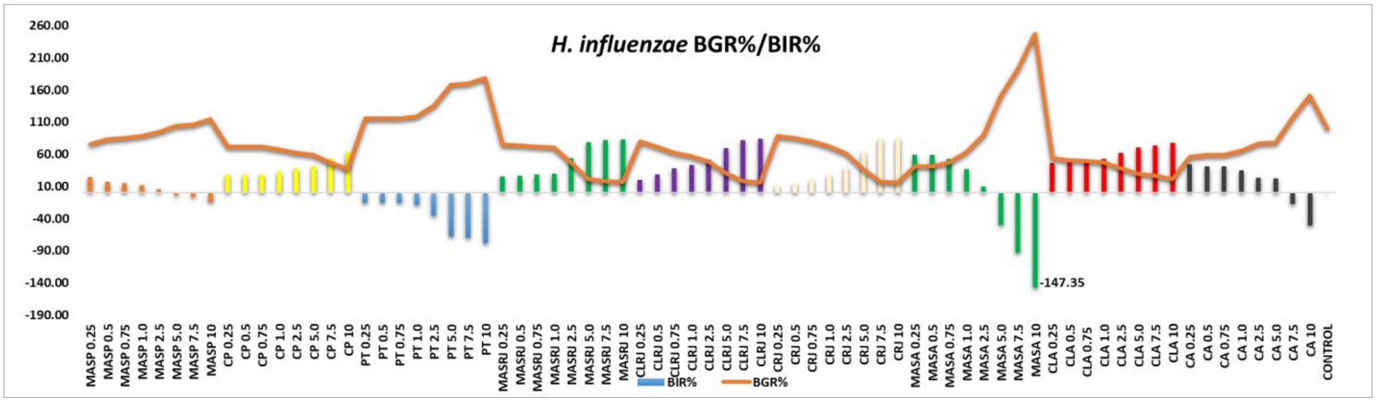

Figure S10. Graphical representation of the BGR% and BIR% values.

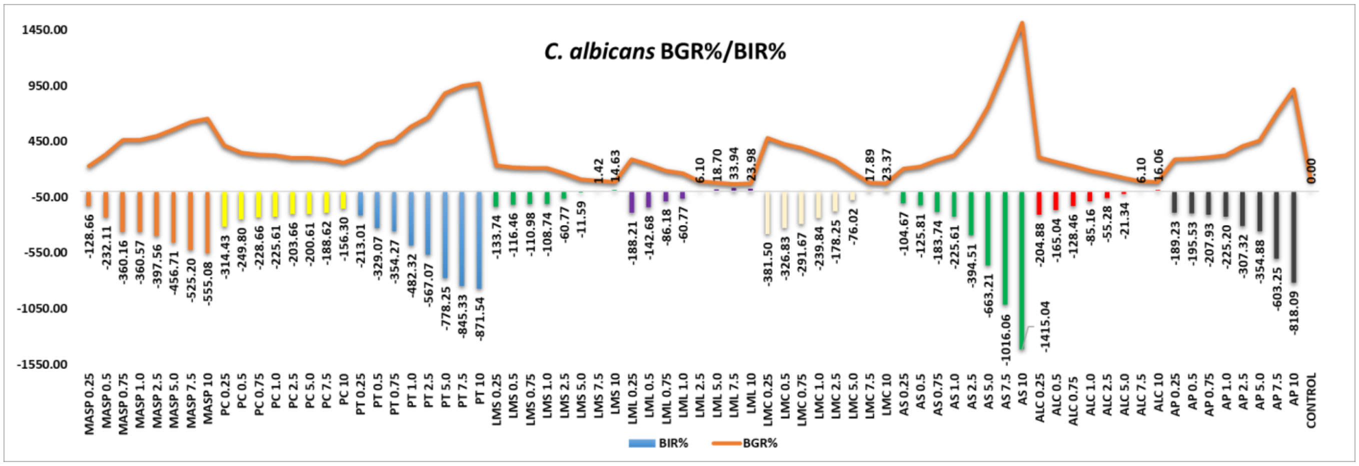

Figure S11. *C. albicans* - Graphical representation of the BGR% and BIR% values.

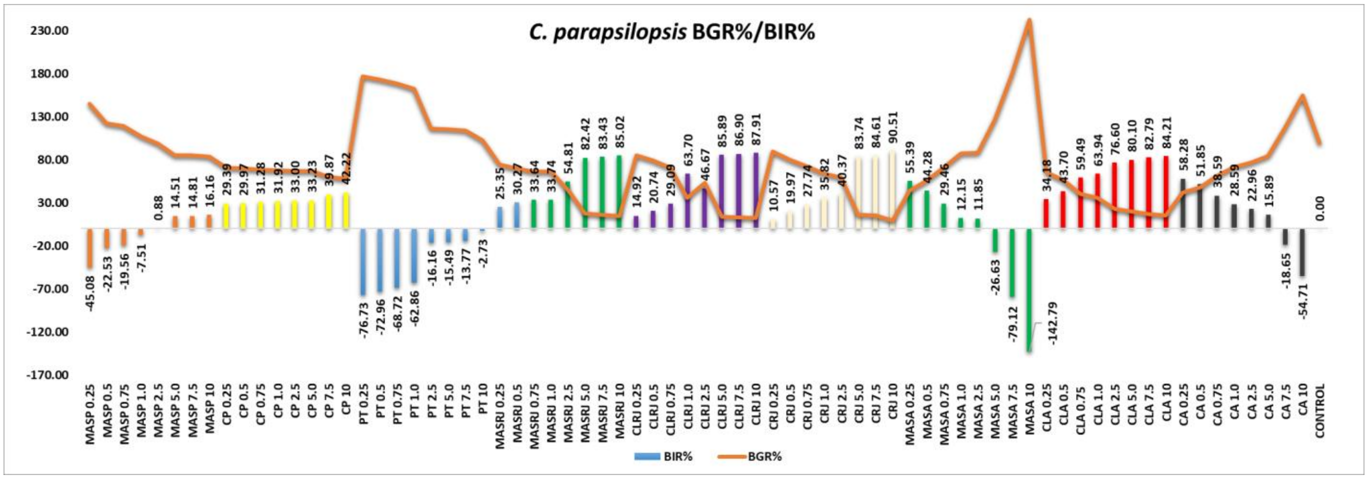

Figure S12. *C. parapsilopsis* - Graphical representation of the BGR% and BIR% values.

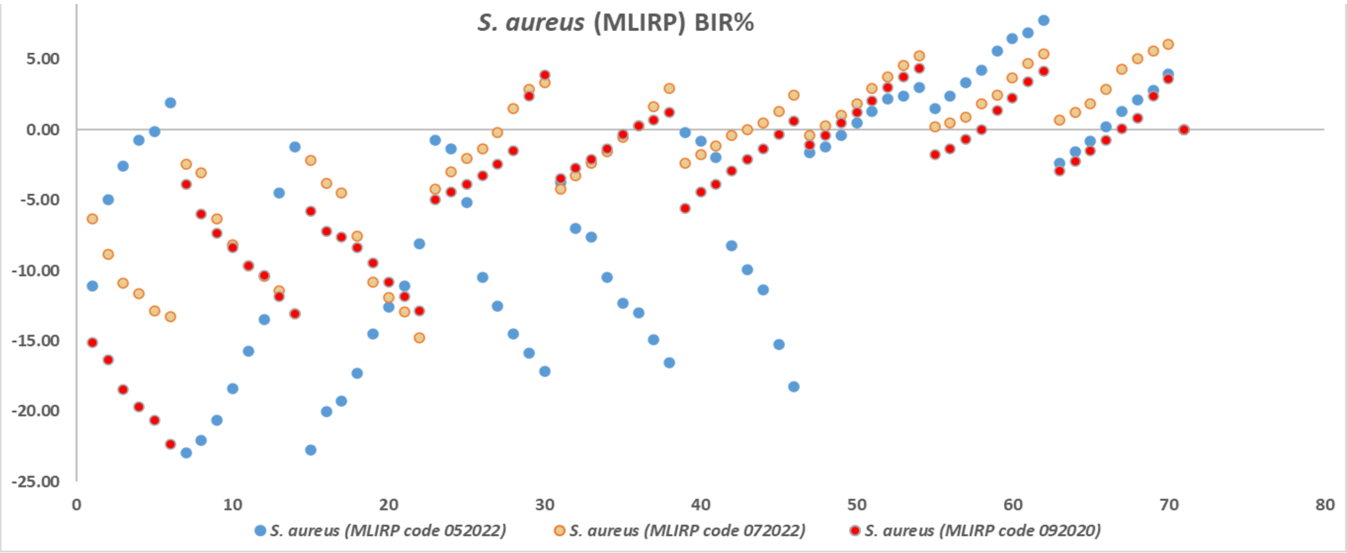

Figure S13. Graphical representation of BIR% of *S. aureus* isolates

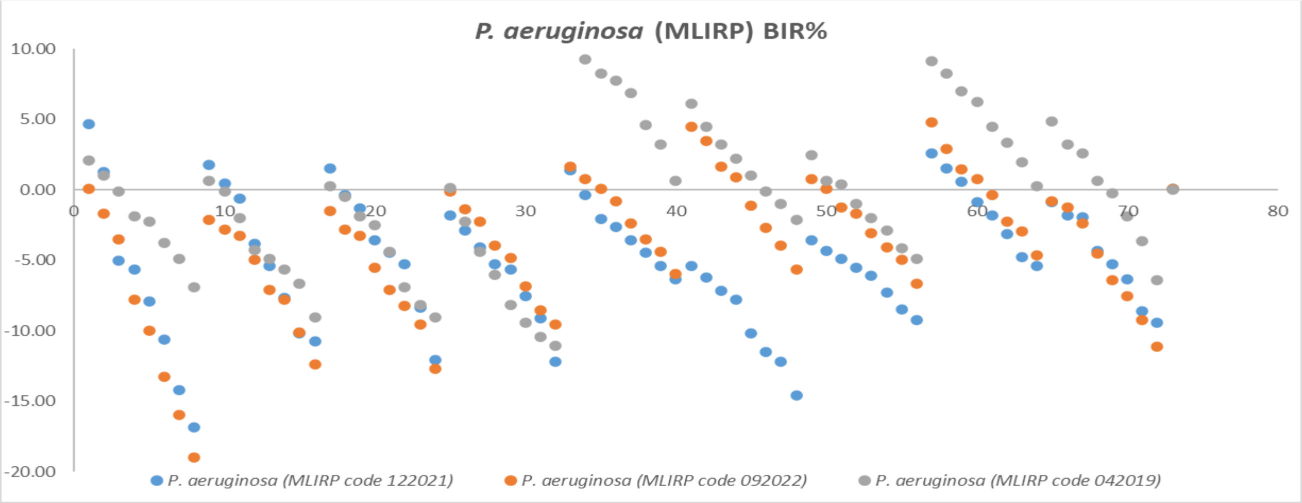

Figure S14. Graphical representation of BIR% of *P. aeruginosa* isolates

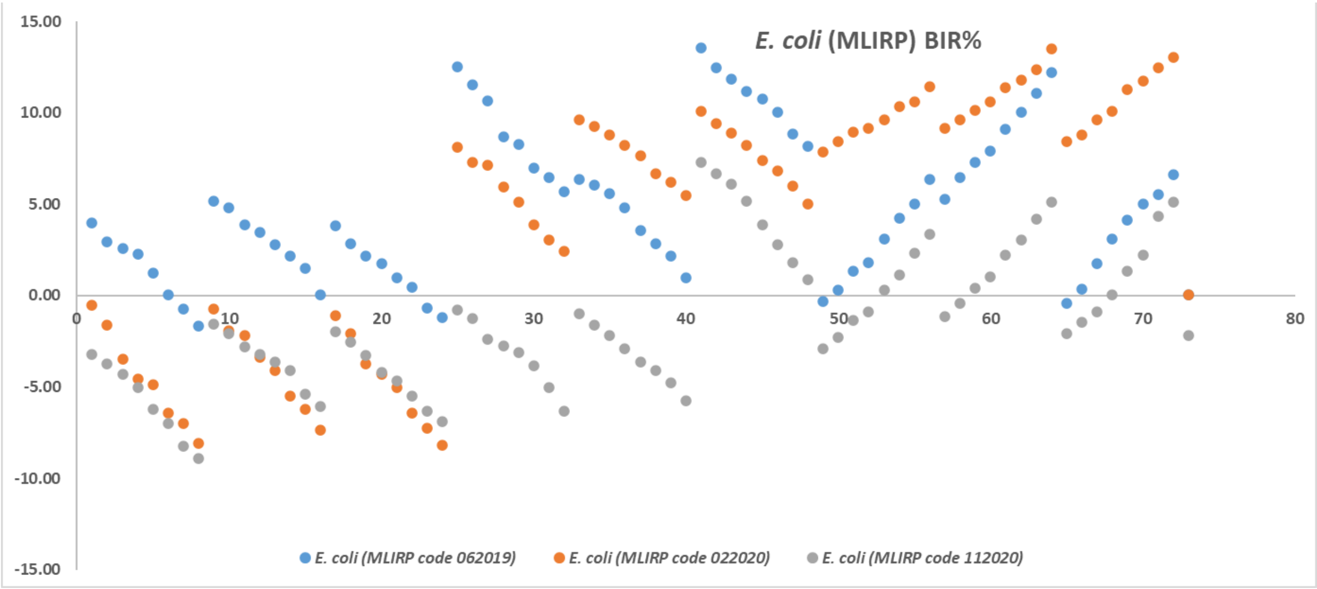

Figure S15. Graphical representation of BIR% of *E.coli* isolates
